# A chicken and egg situation: a case of left bundle branch block-induced dilated cardiomyopathy

**DOI:** 10.1186/s12872-026-05626-x

**Published:** 2026-02-20

**Authors:** Bo Zhang, Fu-Yan Chen, Hua-Zhen Huang, Jin-Long Deng, Dong-Feng Wu

**Affiliations:** 1 Department of Geriatric Cardiology, Guangxi Academy of Medical Sciences and the People’s Hospital of Guangxi Zhuang Autonomous Region, 6 Taoyuan Road, Nanning, Guangxi 530021 China; 2 Key Laboratory of Human Development and Disease Research (Guangxi Medical University), Education Department of Guangxi Zhuang Autonomous Region, 22 Shuangyong Road, Nanning, Guangxi 530021 China

**Keywords:** Left bundle branch block -induced dilated cardiomyopathy, Left bundle branch block, Heart failure, Cardiac resynchronization therapy, Arrhythmogenic cardiomyopathy

## Abstract

**Background:**

Left bundle branch block (LBBB) mainly occurs in those with heart disease, but also appears in asymptomatic patients with normal heart structure. Determining the causal relationship between LBBB and cardiomyopathy is a current clinical challenge.

**Case presentation:**

This article describes a case of a middle-aged female patient with complete LBBB and cardiac dysfunction whose symptoms significantly improved after cardiac resynchronization therapy (CRT). During follow-up assessments at 3 and 6 months postsurgery, echocardiography revealed good recovery of cardiac structure and function, with a significant improvement in cardiac functional classification (NYHA). A comparison of the preoperative and postoperative echocardiographic data revealed that the patient's cardiac structural and functional indicators improved. The patient was ultimately diagnosed with LBBB-induced dilated cardiomyopathy (LIC). Additionally, the potential value of cardiac magnetic resonance (CMR) imaging for predicting the therapeutic effect of CRT on LIC was demonstrated.

**Conclusion:**

Through the analysis of medical history, cardiac ultrasound, and CMR imaging, LIC can be detected early and accurately.

**Supplementary Information:**

The online version contains supplementary material available at 10.1186/s12872-026-05626-x.

## Background

Left bundle branch block (LBBB)-induced dilated cardiomyopathy (LIC) represents a distinct form of cardiomyopathy that is characterized by cardiac remodeling and dysfunction. However, LIC has not been classified under the categories of dilated cardiomyopathy or unclassified cardiomyopathy [1]. The asynchrony of ventricular contraction leads to remodeling of the left ventricle, a reduced ejection fraction, and worsening of clinical symptoms, thereby further deteriorating cardiac function and leading to a poor response to conventional pharmacological treatments. Cardiac resynchronization therapy (CRT) is a therapeutic intervention that aims to increase the mechanical efficiency of the heart and promote blood circulation. This technique facilitates synchronized contraction of the left and right ventricles by implanting a left ventricular electrode within the left ventricular activation zone and a right ventricular electrode lead in the right ventricle, utilizing coronary sinus venography for guidance [2]. CRT is considered the treatment of choice for LIC patients. The early detection of LIC and timely administration of CRT may improve the overall prognosis of this condition. There are relatively few epidemiologic studies on LIC, and it is unclear what proportion of patients with heart failure have this form of cardiomyopathy. Therefore, there is no consensus regarding the diagnosis, treatment, and management of LIC. The response to CRT varies individually among patients, and its effectiveness is influenced by multiple factors, including the severity of LBBB, the presence of ventricular remodeling, and the distribution of myocardial fibrosis. Therefore, the precise assessment of indications and the optimization of patient selection criteria have become important issues in clinical practice.

The aim of this case report was to describe the diagnostic and therapeutic experience of a patient with LIC to help clinicians manage similar cases.

## Case presentation

In February 2024, a 55-year-old female patient was admitted to the People's Hospital of Guangxi Zhuang Autonomous Region (Nanning, China) with recurrent chest tightness and shortness of breath for 3 years as well as recurrent palpitations for 1 month. The patient did not have a history of smoking or alcohol consumption, and she did not have a family history of hypertension, coronary heart disease, cardiomyopathy, or type 2 diabetes mellitus.

In 2021, the patient began to experience recurrent chest tightness and precordial pain, which presented after activity and lasted for more than 10 min. She presented to a local hospital, where an electrocardiogram demonstrated a complete left bundle branch block (LBBB). Subsequent echocardiography and percutaneous coronary angiography did not reveal any significant abnormalities. No medical intervention was deemed necessary, and the patient was discharged following the spontaneous resolution of her symptoms. Subsequently, the patient's symptoms recurred, and she visited the local outpatient clinic at irregular intervals. In November 2023, the aforementioned symptoms reemerged, exhibiting a similar nature to the prior manifestations. Consequently, the patient sought care at a local medical facility where cardiac ultrasonography was conducted. This investigation revealed that the left ventricular end-diastolic dimension (LVDD) was 58 mm (mm, ref: 35–50 mm), the left ventricular end-systolic diameter (LVESD) was 48 mm (ref: 25–35 mm), the overall motion of the ventricular wall was weakened, and the left ventricular ejection fraction (LVEF) was 37%. The patient was prescribed a regimen of oral sacubitril valsartan 100 mg po bid, metoprolol succinate 47.5 mg po qd, spironolactone 20 mg po qd, and dapagliflozin 10 mg po qd to treat heart failure. However, the patient did not adhere to the prescribed schedule for regular follow-up visits. In January 2024, her initial symptoms reemerged and were accompanied by palpitations. ECG revealed a "complete left bundle branch block." Consequently, the patient sought medical attention at our hospital.

**Fig. 1:**
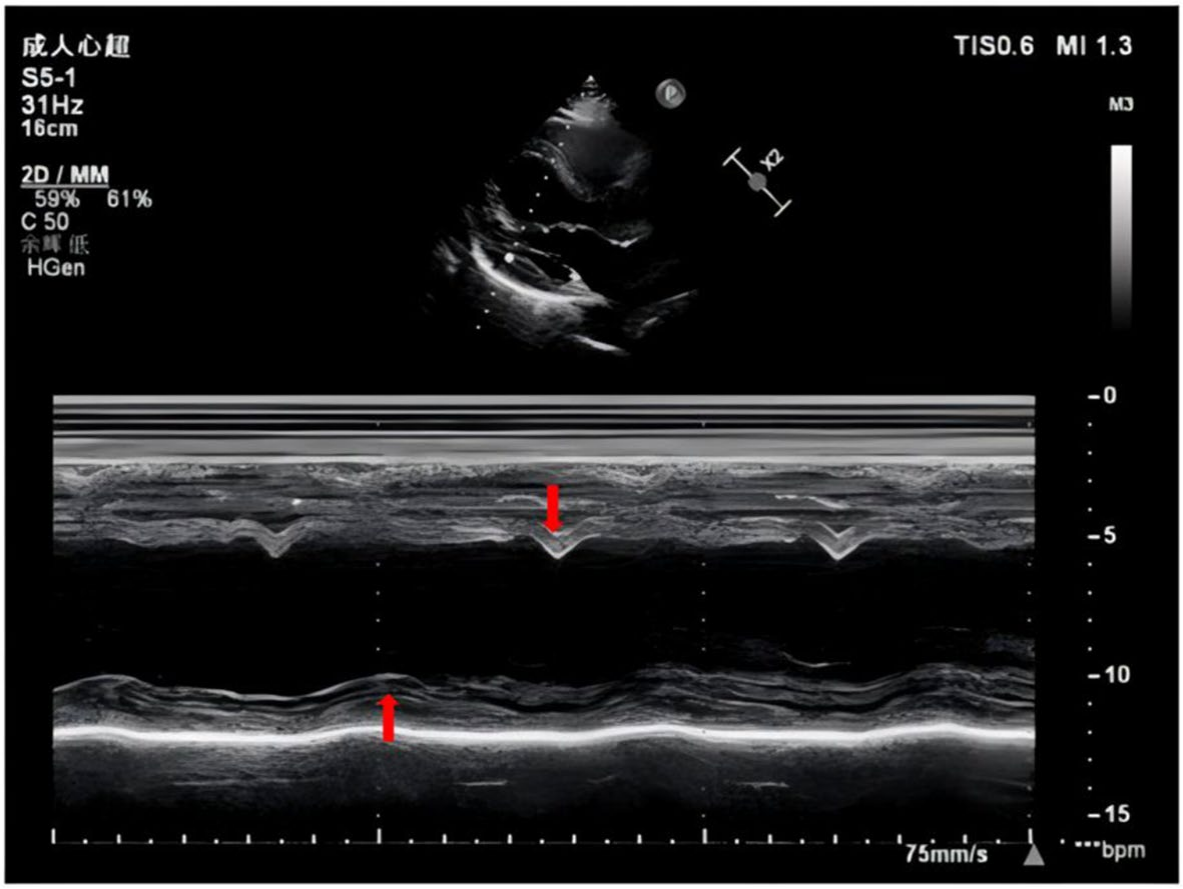
M-mode echocardiography showing inter-ventricular septal–posterior wall motion asynchrony.M-mode echocardiography showing septal–posterior wall motion asynchrony; the vertical bars mark the time difference between peak inward motion of the inter-ventricular septum (IVS) and the posterior wall (PW)

**Fig. 2:**
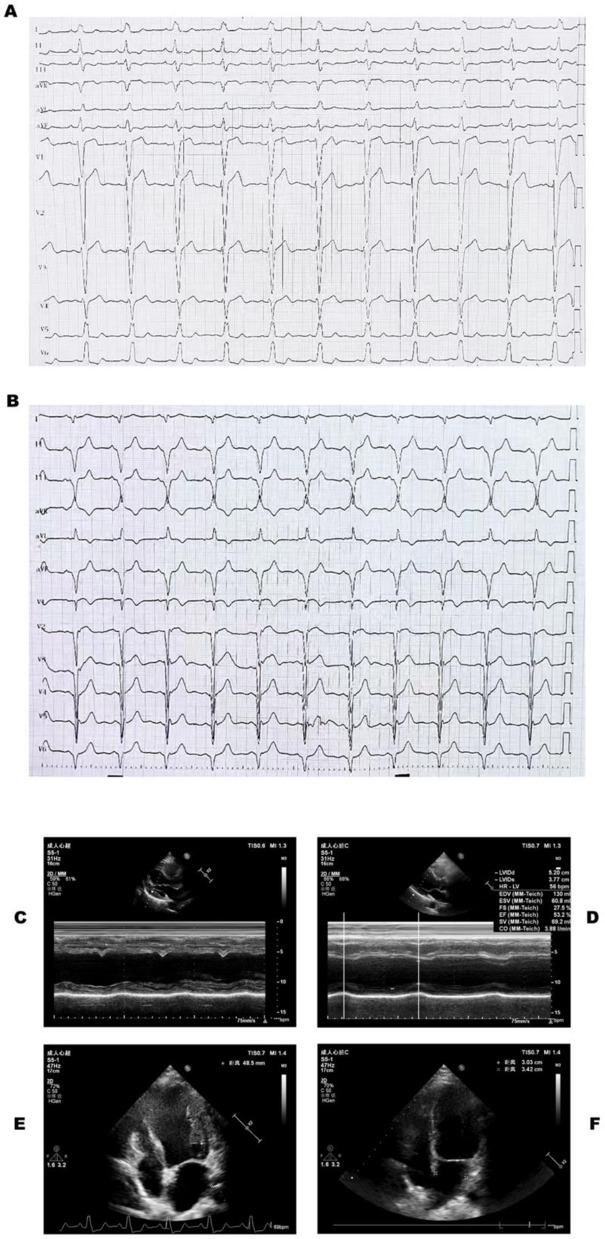
Comparison of ECG and Echocardiography Before and After CRT. **A** Preoperative: ECG shows complete left bundle branch block (LBBB) with a QRS wave duration of 168 ms; **B** Postoperative: QRS wave duration narrowed to 80 ms; **C** Right and left ventricular desynchrony; **D** synchronous contraction. **E** and **F** indicate echocardiographic comparisons of ventricular reverse remodeling in patients before and after CRT, respectively

A cardiac examination revealed slightly enlarged heart boundaries, synchronized heart rhythm, and the absence of significant heart murmurs in the auscultation area of the individual valves of the heart. No edema was observed in either lower extremity. The cardiac enzyme profile was found to be within normal limits. ECG revealed sinus rhythm and complete left bundle branch block with a QRS wave duration of 168 ms (Fig. 2A), concurrently fulfilling the following criteria: 1. An rS pattern in lead V1; 2. A unidirectional broad R wave in lead V6, accompanied by ST-T segment changes that are discordant to the direction of the main QRS complex; 3. Absence or minimal presence of Q waves in leads I, aVL, V5, and V6. This aligns with the diagnosis of a 'true' left bundle branch block (LBBB) as per the Strauss criteria. Echocardiography (Fig. 1) revealed that the left atrial anteroposterior diameter (LAD-ap) was 34 mm (ref: 20–35 mm), and the right and left diameters were 49 mm. The LVDD was 61 mm (ref: 35–52 mm), and the LVESD was 56 mm (ref: 20–35 mm), with the overall motion of the ventricular wall being weakened and poorly coordinated. The LVEF was 26%, and the cardiac output (CO) was 3.7 L/min, suggesting myocardial involvement. The cardiac magnetic resonance imaging (CMR) revealed left ventricular dilatation accompanied by global systolic dysfunction (EF26.4%). Multiple mid-wall fibrotic patches were observed in the inferior and antero-lateral walls, as well as in the mid-septum. Additionally, septal flash, apical rocking, which is indicative of dyssynchrony associated with left bundle branch block (see Video 1Video 1.mp4). The preliminary diagnosis was cardiomyopathy (LIC?) and heart failure NYHA class II.

The patient has no history of hypertension, as evidenced by multiple blood pressure measurements consistently below 130/80 mmHg, thereby excluding the possibility of hypertensive heart disease. Coronary angiography did not reveal any significant stenosis, effectively ruling out ischemic cardiomyopathy. CMR demonstrated left ventricular enlargement accompanied by reduced systolic function. Multiple mid-wall line-like delayed enhancement lesions were identified in the inferior wall, mid-proximal segments of the lateral wall, and mid-septum, which are indicative of non-ischemic, non-replacement fibrosis, yet are still considered stress-induced fibrotic changes. The synchronous contraction of the septum and right ventricular free wall suggested mechanical dyssynchrony attributable to left bundle branch block. In conjunction with echocardiographic findings of "large cavities with thin walls, diffuse hypokinesis, and dyssynchrony," these observations collectively support a definitive diagnosis of LIC. Given the patient's chronic heart failure and her LVEF of less than 35%, the optimal medical therapy was administered for three months. However, no significant improvement in LVEF was noted, and the QRS duration remained ≥ 150 ms. With an anticipated survival period exceeding one year and heart failure NYHA class II, there was a clear indication for CRT via the implantation of a biventricular resynchronization pacemaker in conjunction with an ICD. After ruling out any surgical contraindications, CRT was carried out on February 27, 2024.

The postoperative electrocardiogram (Fig. 2B) revealed that sinus rhythm and the QRS duration narrowed to 80 ms. Echocardiography confirmed the correct positioning of the pacemaker within the atrioventricular cavities, and the LVEF was 35%.

Following the amelioration of the patient's symptoms, the patient was discharged and prescribed an exercise rehabilitation regimen. The patient subsequently attended outpatient follow-up appointments at 3 months and 6 months after surgery. Table 1 shows the differences in the patient's echocardiography parameters. Relative to the presurgical state, the patient's symptoms, cardiac function, and overall heart condition exhibited marked recovery, thus enabling her to essentially manage routine physical activities.

**Table 1:** Changes in echocardiography parameters

Time	LA-ap (mm)	LA-t (mm)	LVDD (mm)	LVESD (mm)	LVEF	CO (L/min)	Biventricular pacing (%)	NYHA functional class
2024–2(baseline)	34	49	61	56	26%	3.7	/	Ⅱ
2024–3 (post-CRT)	29	31	47	37	35%	2.5	/	Ⅱ
2024–6 (after 3 m)	35	31	52	40	45%	3.7	98.1	Ⅰ
2024–9 (after 6 m)	31	34	52	38	53%	3.9	99.3	Ⅰ

Reference range: LA-ap, 20–35 mm; LA-t, 2–35 mm; LVDD, 35–52 mm; LVESD, 20–35 mm

*NYHA* New York Heart Association, *LA-t* left atrial transverse diameter

## Discussion and conclusions

The patient was a middle-aged female. In 2021, complete LBBB was detected via electrocardiography, while an echocardiogram indicated normal cardiac function. The time window for the discovery of left ventricular dysfunction was only 3 years, and other potential causes, such as ischemic cardiomyopathy, were ruled out. After receiving CRT, the patient achieved a CRT superresponse within 6 months, thus confirming the diagnosis of LIC.

LBBB primarily manifests in individuals with underlying heart disease; however, its occurrence is not uncommon in asymptomatic patients with a structurally normal heart. Recent research has emphasized the pivotal role of CRT in the management of patients with LBBB and severe heart failure1. However, clinical studies have revealed that approximately 25% of patients fail to respond to CRT, with a subset of patients even experiencing exacerbation of heart failure symptoms postimplantation [3]. Ongoing clinical challenges include determining the causal nexus between LBBB and cardiomyopathy as well as predicting the efficacy of CRT. In this context, the identification of a reliable clinical indicator is highly important. CMR imaging has emerged as an increasingly significant tool because of its specificity in diagnosing cardiomyopathy. On CMR imaging, patients with LIC exhibit preserved lateral wall function and morphology, distinguished by greater lateral wall thickness, an elevated lateral wall thickening rate, and a greater ratio of lateral wall to septal thickness than those with cardiomyopathy stemming from other causes [4]. Notably, when LBBB is secondary to other cardiomyopathies, CRT may correct the electromechanical desynchrony of the ventricles without halting the progression of the underlying cardiomyopathy, potentially resulting in suboptimal CRT outcomes. LIC is primarily attributed to cardiac electromechanical desynchrony, leading to cardiac enlargement and heart failure. Following CRT, which rectifies the mechanical desynchrony between the left and right ventricles, patients often experience substantial symptomatic improvement, with some patients even achieving normalization of cardiac function [5].

In the current case report, following the detection of asynchronous movement between the left and right ventricles via echocardiography, CMR imaging was conducted. The results indicated a weakened overall contraction of the left ventricle, particularly affecting the septum. The septal movement was found to be uncoordinated; however, it was synchronized with the contraction of the right ventricular free wall. Notably, the function and structure of the ventricular lateral wall remained well preserved. By integrating the patient's medical history, ECG, EF, and characteristics of CMR imaging, it was concluded that the patient's LBBB preceded the decline in EF. CMR imaging also indicated good preservation of ventricular structure and function, leading to a preoperative diagnosis of LIC. Six months of post-CRT implantation follow-up revealed an ultraresponse to CRT. Consequently, through the analysis of medical history, cardiac ultrasound, and CMR imaging, LIC can be detected early and accurately, potentially reducing the number of unnecessary CRT implantations. Furthermore, the ventricular wall thickening observed via CMR imaging may serve as a predictive marker for CRT response and treatment outcomes for LIC.

Currently, the guidelines for CRT in patients with LIC are derived primarily from recommendations for CRT in patients with dilated cardiomyopathy and LBBB. There are no specialized guidelines tailored specifically for LIC. Nonetheless, in clinical settings, there is a debate over whether patients who are superresponders to CRT actually have LIC rather than dilated cardiomyopathy with LBBB. Recent research efforts have predominantly focused on retrospective clinical analyses; however, the sample sizes are limited, and the data available are insufficient. Consequently, randomized controlled trials will be essential for validating our hypothesis and distinguishing these patients, thereby reducing the occurrence of nonresponse to CRT.

This case report detailed the complete disease course of a typical LIC patient, from the electrocardiographic changes in LBBB through cardiac dysfunction, treatment, and eventual recovery. To date, LIC remains a diagnosis that necessitates further investigation to exclude cardiac dilation as a potential etiology. Nevertheless, CMR imaging will serve as a crucial reference for both diagnostic and therapeutic purposes. However, late gadolinium enhancement lesion and LBBB lack a direct relationship and demand further in-depth investigation. This report will serve as a valuable reference for clinicians managing similar cases.

## Supplementary Information

Supplementary Material 1.

## Data Availability

The original contributions presented in the study are included in the article. Further inquiries can be directed to the corresponding authors.
